# Revision Lumbar Spine Surgery for Failed Back Surgery Syndrome: A Retrospective Study of Causes, Complications, and Outcomes

**DOI:** 10.7759/cureus.113043

**Published:** 2026-07-20

**Authors:** Ahmed Al Atraqchi, Maher Nazar, Rawan Alatraqchi, Yasoob A Alaameri

**Affiliations:** 1 Neurological Surgery, Dr. Saad Al-Witry Neuroscience Hospital, Baghdad, IRQ; 2 Emergency Medicine, Calderdale and Huddersfield NHS Foundation Trust, Halifax, GBR; 3 Neurological Surgery, Baghdad Medical City, Baghdad, IRQ

**Keywords:** failed back surgery syndrome, incomplete decompression, oswestry disability index, pseudoarthrosis, recurrent disc herniation, revision lumbar surgery, visual analogue scale

## Abstract

Background: Failed back surgery syndrome (FBSS) describes persistent, recurrent, or new-onset low back pain and/or radicular symptoms following lumbar spine surgery. Revision lumbar surgery remains challenging because of altered anatomy, epidural scarring, unclear tissue planes, and a higher risk of complications. Careful identification of a surgically correctable pathology is essential to improve outcomes.

Aim: The aim of this study was to evaluate the causes of failed previous lumbar surgery, indications for revision surgery, operative procedures performed, postoperative complications, and clinical and functional outcomes in patients undergoing revision lumbar spine surgery for FBSS.

Methods: This retrospective observational study included 150 patients who underwent revision lumbar spine surgery for FBSS between October 2017 and September 2023, with follow-up completed until September 2025. Patients were included if they had persistent or recurrent symptoms after previous lumbar surgery with radiological evidence of a surgically correctable pathology. Clinical data, operative findings, radiological diagnoses, complications, and follow-up outcomes were reviewed. Functional outcomes were assessed using the Visual Analogue Scale (VAS) and Oswestry Disability Index (ODI) preoperatively and at three, 12, and 24 months postoperatively. Statistical analysis was performed using IBM SPSS Statistics for Windows, version 29 (IBM Corp., Armonk, New York, United States), with p < 0.05 considered statistically significant.

Results: The study included 84 male and 66 female patients, with a mean age of 41 ± 10.09 years. The most common presenting symptom was sciatica, reported in 63 patients (42%), followed by low back pain with sciatica in 45 patients (30%). The most frequently revised level was L4/L5 in 48 patients (32%). Incomplete decompression was the most common cause of failed previous lumbar surgery, identified in 42 patients (28%), followed by recurrent disc herniation in 39 patients (26%), retained disc fragment in 36 patients (24%), pseudoarthrosis or instrumentation-related failure in 24 patients (16%), and wrong-level surgery in nine patients (6%). Perioperative complications occurred in 69 patients (46%), with dural tear being the most common complication in 24 patients (16%). At three months, 93 patients (62%) were pain-free, increasing to 105 patients (70%) at 12 months, and maintained at 24 months. Sensory improvement occurred in 15 of 48 patients (31%), while motor improvement occurred in 21 of 39 patients (53%). The mean VAS score improved from 7.22 ± 0.89 preoperatively to 0.98 ± 0.75 at 24 months, and the mean ODI improved from 66.30 ± 9.06 to 7.77 ± 3.55. Improvements in both VAS and ODI were statistically significant (p = 0.001).

Conclusion: This study provides long-term evidence that revision lumbar surgery can achieve sustained improvements in pain and disability over 24 months in carefully selected patients with a surgically correctable pathology. It also demonstrates that potentially preventable technical factors, particularly incomplete decompression and retained disc fragments, represent important causes of failed previous surgery. These findings support the importance of accurate clinical-radiological correlation, careful patient selection, and meticulous surgical planning.

## Introduction

Failed back surgery syndrome (FBSS) describes persistent, recurrent, or new-onset low back and/or lower-extremity pain following one or more spinal surgical procedures. For the purposes of this study, symptoms persisting or recurring for at least three months after surgery were considered chronic, in accordance with the International Association for the Study of Pain definition of chronic postsurgical pain [1,2].

Although FBSS is not a single pathological diagnosis, it remains clinically important because it represents a complex postoperative condition in which pain may arise from structural, mechanical, neurological, or psychosocial factors [3,4]. The causes of FBSS are multifactorial and may be related to preoperative, operative, or postoperative factors. Preoperative factors include poor patient selection, inadequate clinical-radiological correlation, psychosocial comorbidity, and inappropriate surgical planning. Operative causes include wrong-level surgery, inadequate decompression of the lateral recess or neural foramina, excessive decompression leading to instability, or incomplete removal of compressive pathology. Postoperative causes include recurrent or retained disc herniation, epidural fibrosis, adjacent segment degeneration, pseudoarthrosis, instrumentation-related failure, nerve root entrapment, and progression of degenerative disease [5,6].

Clinical assessment remains the first step in evaluating patients with suspected FBSS. The pattern of pain, neurological deficits, timing of symptom recurrence, and response to the primary operation may provide important clues regarding the underlying pathology. Early persistence of radicular symptoms may suggest inadequate decompression, retained disc fragment, or wrong-level surgery, while recurrence after a symptom-free interval may suggest recurrent disc herniation, adjacent segment disease, instability, or pseudoarthrosis. Conservative treatment, including analgesia, physiotherapy, rehabilitation, and multidisciplinary pain management, is usually considered before revision surgery, particularly when there is no progressive neurological deficit or clearly correctable structural lesion [7].

Imaging plays a central role in identifying the cause of persistent or recurrent symptoms after lumbar surgery. Magnetic resonance imaging (MRI) with contrast is commonly used to evaluate recurrent disc herniation, residual or recurrent stenosis, nerve root compression, epidural fibrosis, and adjacent segment disease. Computed tomography (CT) is particularly useful in patients with previous instrumentation or suspected bony pathology, including pseudoarthrosis, screw loosening, screw malposition, or inadequate bony decompression. CT myelography may also be useful when MRI is contraindicated, limited by artefact, or insufficient to explain the patient’s symptoms [8].

The number of lumbar fusion procedures has increased over time, and this has been accompanied by an increasing need for revision lumbar surgery. Revision surgery is technically more demanding than primary surgery because of altered anatomy, epidural scarring, distorted tissue planes, adhesions around neural structures, and the possible presence of previous instrumentation. These factors may increase operative time, blood loss, and the risk of complications, including dural tear, cerebrospinal fluid leakage, wound infection, neurological deficit, and persistent pain [9,10]. Despite these challenges, revision surgery may provide meaningful clinical improvement when patients are carefully selected and when the operation is directed toward a clearly identified surgically correctable pathology. Surgical options may include repeat discectomy, extended decompression, foraminotomy, adhesiolysis, revision instrumentation, or fusion, depending on the cause of failure. The choice of procedure should be individualised according to the underlying pathology, spinal stability, previous procedure, radiological findings, and patient-related factors [11,12].

Data on FBSS and revision lumbar surgery from Middle Eastern populations remain relatively limited. Regional studies are important because patient demographics, patterns of referral, access to advanced imaging, availability of spinal instrumentation, surgical techniques, and postoperative rehabilitation pathways may differ between healthcare systems. Further regional data are therefore valuable to better understand the causes of failed lumbar surgery, operative challenges, complication patterns, and clinical outcomes in local neurosurgical practice. The primary objective of this study was to evaluate the clinical and functional outcomes of revision lumbar spine surgery for FBSS. The secondary objectives were to identify the causes of failed previous lumbar surgery, describe the indications and operative procedures used for revision surgery, assess perioperative complications, and examine the association between the type of previous surgery and the subsequent cause of failure. We hypothesised that revision surgery would result in significant improvements in pain and disability in carefully selected patients.

## Materials and methods

This was a retrospective single-centre observational study conducted at Dr Saad Al-Witry Neuroscience Hospital, Baghdad, Iraq. All procedures were performed under the supervision of one consultant neurosurgeon. The study was reviewed by the Research Ethics Committee of Dr Saad Al-Witry Neuroscience Hospital/Al Risafa Health District, and formal ethical approval was waived because this was a retrospective review of anonymised routinely collected clinical data (approval number: LREC/DSWN/2026/003). All surgical procedures were performed as part of routine clinical care, and written informed consent for surgery was obtained from all patients before the primary and revision procedures. No additional intervention was performed for the purpose of this study, and patient confidentiality was maintained throughout data collection and analysis.

Study population

Patients who underwent revision lumbar spine surgery between October 2017 and September 2023 and completed postoperative follow-up until September 2025 were included. This ensured that all patients had adequate follow-up for assessment of clinical and functional outcomes up to 24 months. A total of 150 patients with FBSS who underwent revision lumbar surgery were included. Patients were identified from hospital records, operative notes, outpatient follow-up records, and radiological databases. All included patients had clinical symptoms supported by radiological evidence of a surgically correctable pathology.

Inclusion Criteria

Patients were included if they had previous lumbar spine surgery for degenerative lumbar pathology, including laminectomy, foraminotomy, discectomy, or lumbar fusion with or without instrumentation, persistent or recurrent low back pain, radiculopathy, neurogenic claudication, sensory deficit, motor weakness, or a combination of these symptoms, radiological evidence of a surgically correctable cause of failed surgery, revision lumbar surgery performed during the study period, and available preoperative, operative, radiological, and follow-up data. Surgically correctable causes included recurrent disc herniation, retained disc fragment, wrong-level surgery, incomplete decompression, inadequate foraminal decompression, adjacent segment stenosis, postoperative instability, spondylolisthesis, pseudoarthrosis, screw loosening, screw malposition, or instrumentation failure.

Exclusion Criteria

Patients were excluded if they had severe or uncontrolled psychiatric disorders, including active psychosis, severe major depressive disorder with suicidal risk, bipolar disorder with an active manic episode, or significant cognitive impairment affecting reliable pain assessment, informed consent, or follow-up. Chronic pain syndrome unrelated to lumbar pathology, arachnoiditis as the main cause of symptoms, non-structural pain without concordant radiological abnormality, spinal tumour, trauma, active infection as the primary diagnosis, or incomplete clinical or follow-up data.

Definitions

Revision procedures included laminectomy, foraminotomy, discectomy, and fusion with or without instrumentation, depending on the underlying pathology, number of affected levels, and intraoperative findings. The study evaluated the underlying causes of failed previous lumbar surgery, indications for revision surgery, operative procedures performed, postoperative complications, and clinical and functional outcomes. FBSS was defined as persistent, recurrent, or new-onset low back pain and/or radicular symptoms following previous lumbar spine surgery, with failure to achieve satisfactory clinical improvement after the primary operation. Symptoms were considered persistent or recurrent if they lasted or reappeared at least three months after the primary lumbar surgery.

Preoperative assessment

All patients underwent detailed clinical and neurological assessment before revision surgery. Recorded variables included age, sex, presenting symptoms, neurological deficits, type of previous lumbar surgery, operated spinal level, interval between the primary operation and recurrence or persistence of symptoms, and duration of recurrent or persistent symptoms. Presenting symptoms were classified as sciatica, low back pain with sciatica, claudicating sciatica, or low back pain with claudicating sciatica. Neurological deficits were recorded separately and included sensory deficit and motor weakness. Neurological examination included assessment of sensory impairment, motor power, reflexes, gait disturbance, straight leg raising, and sphincter symptoms when present.

The interval between the primary operation and recurrence or persistence of symptoms was categorised as less than 15 months, 15-24 months, or more than 24 months. All patients underwent preoperative imaging to identify the structural cause of symptoms and guide surgical planning. Plain lumbar radiographs were used to assess the level of previous surgery, spinal alignment, and degenerative changes. Dynamic flexion-extension radiographs were performed when instability was suspected. Contrast-enhanced MRI was used to assess recurrent disc herniation, retained disc fragment, residual or recurrent canal stenosis, foraminal stenosis, nerve root compression, epidural fibrosis, and adjacent segment disease. CT was performed particularly in patients with previous instrumentation or suspected bony pathology, including screw loosening, screw malposition, pseudoarthrosis, or inadequate previous decompression. The final diagnosis was made by correlating clinical findings with radiological abnormalities.

Classification of pathology and previous surgery

The cause of FBSS was classified as recurrent disc herniation, retained disc fragment, wrong-level surgery, incomplete decompression, pseudoarthrosis or instrumentation-related failure, postoperative instability or spondylolisthesis, or adjacent segment stenosis. Previous lumbar procedures were classified as microdiscectomy, interlaminar decompression/fenestration, laminectomy, laminectomy with discectomy, or laminectomy with fusion/instrumentation. The relationship between the type of previous procedure and the underlying cause of failure was analysed.

Surgical decision-making and operative procedures

Revision surgery was performed only when symptoms, neurological findings, and imaging demonstrated a concordant surgically correctable pathology. Patients with recurrent or retained disc herniation without instability underwent revision discectomy, hemilaminotomy, fenestration, or epidural adhesiolysis as required. Patients with residual canal stenosis, foraminal stenosis, incomplete decompression, or adjacent segment stenosis underwent decompressive laminectomy, foraminotomy, or extended decompression. Patients with instability, spondylolisthesis, pseudoarthrosis, or instrumentation-related failure underwent decompression with fixation and fusion. Fusion procedures included posterolateral fusion, posterior lumbar interbody fusion, or transforaminal lumbar interbody fusion, depending on the pathology, spinal level, and surgeon preference. Local autologous bone graft harvested from the excised lamina was used for fusion. In patients with previous instrumentation, revision included assessment of screw position, removal or replacement of loose or malpositioned screws, decompression of affected neural elements, and fusion when indicated.

Outcome assessment

Patients were followed postoperatively through inpatient records and outpatient clinic visits. Clinical outcomes included pain improvement, sensory improvement, motor improvement, persistence or recurrence of symptoms, patient satisfaction, and perioperative complications. Functional outcomes were assessed using the Visual Analogue Scale (VAS) for pain [13] and the Oswestry Disability Index (ODI) [14], which was used with permission. VAS and ODI scores were obtained using patient-reported questionnaires during routine preoperative and postoperative follow-up visits, documented in the medical records, and retrospectively extracted for analysis. Preoperative scores were compared with postoperative scores at three, 12, and 24 months. Perioperative complications included intraoperative dural tear and neurological injury, as well as postoperative wound infection, discitis, pseudoarthrosis, and instrumentation-related complications. The original descriptions of the assessment tools were cited accordingly. 

Statistical analysis

Statistical analysis was performed using IBM SPSS Statistics for Windows, version 29 (IBM Corp., Armonk, New York, United States). Continuous variables were expressed as mean ± standard deviation (SD), while categorical variables were expressed as frequencies and percentages. The relationship between the type of previous lumbar surgery and the underlying cause of FBSS was analysed using the chi-square test of independence. Fisher’s exact test was used when expected cell counts were less than 5. Changes in VAS and ODI scores across preoperative, three-month, 12-month, and 24-month follow-up assessments were analysed using repeated-measures analysis of variance. A p-value of less than 0.05 was considered statistically significant.

## Results

Patient demographics and clinical presentation

A total of 150 patients underwent revision lumbar surgery for FBSS during the study period. There were 84 male and 66 female patients, giving a male-to-female ratio of 1.27:1. Most patients were aged between 40 and 60 years (n=84, 56%). Forty-five patients (30%) were older than 60 years, while 21 (14%) were younger than 40 years. The mean age was 41 ± 10.09 years. The demographic characteristics, including age group and sex distribution, are summarised in Table 1.

**Table 1:** Demographic characteristics of study population (N=150)

Age group (years)	Sex		Total patients, n (%)
	Female (n=66), n	Male (n=84), n	
< 40	12	9	21 (14%)
40 - 60	36	48	84 (56%)
> 60	18	27	45 (30%)

**Figure 1:**
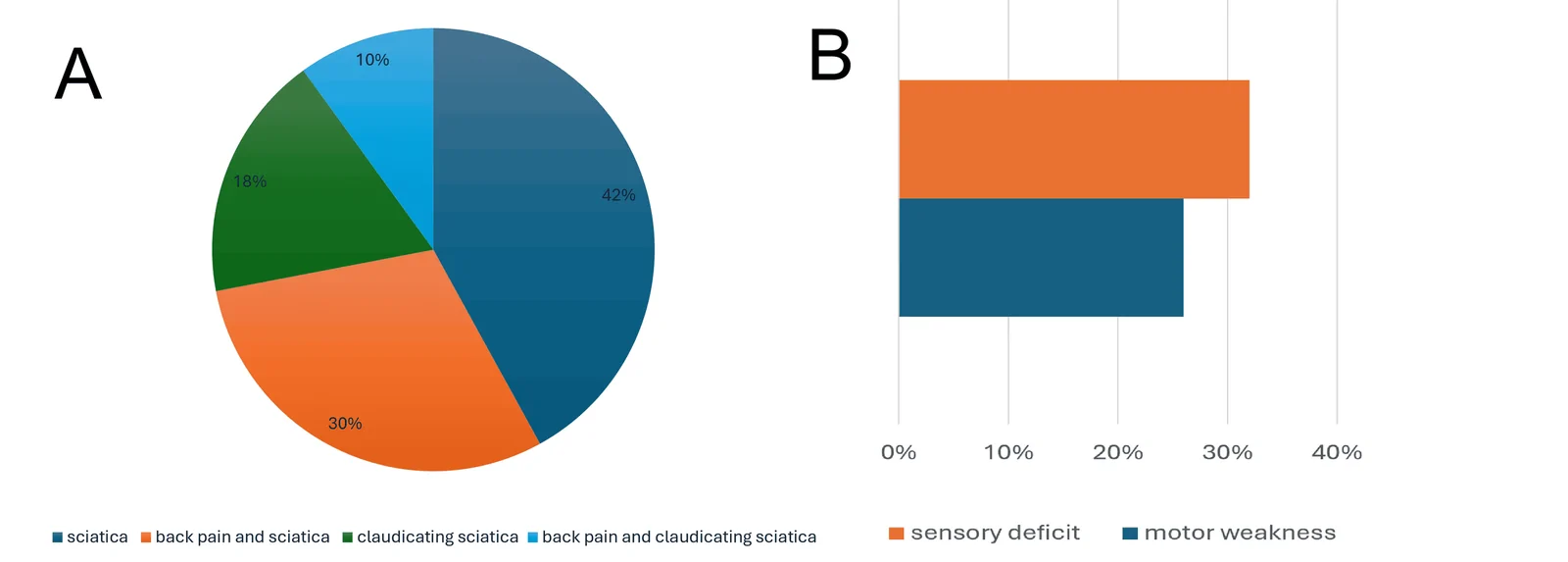
Clinical presentation of study population (A) Distribution of presenting symptoms. (B) Frequency of neurological deficits.

The most common presenting symptom was sciatica, reported in 63 patients (42%). Low back pain with sciatica was present in 45 patients (30%), claudicating sciatica in 27 patients (18%), and low back pain with claudicating sciatica in 15 patients (10%). The presenting symptoms identified in this cohort are shown in Figure 1A. Neurological deficits were also observed (Figure 1B); 48 patients (32%) had sensory deficits and 39 (26%) had motor weakness. 

Interval between primary and revision surgery

The interval between the primary lumbar operation and recurrent or persistent symptoms varied among patients. Symptoms recurred or persisted within less than 15 months in nine patients (6%), between 15 and 24 months in 37 patients (25%), and after more than 24 months in 104 patients (69%) (Table 2). Nine patients underwent revision surgery for the third time (data not shown).

**Table 2:** Interval between primary lumbar surgery and recurrent/persistent symptoms (N=150)

Interval	Number of Patients	Percentage
Less than 15 months	9	6%
15-24 month	37	25%
More than 24 months	104	69%

Operated spinal levels

The most frequently revised spinal level was L4/L5, involved in 48 patients (32%). This was followed by L3/L4 (n=27, 18%), and L5/S1 (n=21, 14%). Multilevel surgery was performed in 54 patients. Combined L3/L4 and L4/L5 surgery was performed in nine patients (6%), combined L4/L5 and L5/S1 surgery in 18 patients (12%), and three-level L3/L4, L4/L5, and L5/S1 surgery in 27 patients (18%). The findings are summarised in Table 3.

**Table 3:** Operated spinal levels at revision surgery (N=150)

level of operation	Number of Patients	Percentage
L3/4	27	18%
L4/5	48	32%
L5/S1	21	14%
L3/4 and L4/5	9	6%
L4/5 and L5/S1	18	12%
L3/4 and L4/5 and L5/S1	27	18%

Causes of failed previous lumbar surgery

The underlying causes of FBSS included recurrent disc herniation, retained disc fragment, wrong-level surgery, incomplete decompression, and pseudoarthrosis or instrumentation-related failure. Incomplete decompression was the most frequent cause, identified in 42 patients (28%). Recurrent disc herniation was identified in 39 patients (26%), retained disc fragment in 36 patients (24%), pseudoarthrosis or instrumentation-related failure in 24 patients (16%), and wrong-level surgery in nine patients (6%).

When the cause of failure was analysed according to the type of previous surgery, recurrent disc herniation was most frequently associated with previous microdiscectomy and interlaminar decompression/fenestration. Among patients who had previous microdiscectomy, 18 of 42 patients had recurrent disc herniation, 12 had retained disc fragments,six had wrong-level surgery, and six had pseudoarthrosis or instrumentation-related failure. Among patients who had previous interlaminar decompression/fenestration, 12 of 30 patients had recurrent disc herniation, nine had retained disc fragments, three had wrong-level surgery, three had incomplete decompression, and three had pseudoarthrosis or instrumentation-related failure. Incomplete decompression was most observed after previous laminectomy or laminectomy with fusion. Of 24 patients with previous laminectomy and 27 patients with previous laminectomy and fusion, 18 and 21, respectively, had incomplete decompression.

Among patients who had previous laminectomy with discectomy, retained disc fragment was the most common cause of failure, occurring in 15 of 27 patients, followed by recurrent disc herniation in nine patients and pseudoarthrosis or instrumentation-related failure in three patients. Residual disc herniation was defined as persistent disc material associated with no satisfactory postoperative improvement, whereas recurrent disc herniation was defined as a new herniation after an initial period of symptomatic improvement. The correlation between previous surgery and diagnosis is demonstrated in Table 4.

**Table 4:** Correlation between previous surgery and diagnosis

Previous surgeries	Recurrent disc (n=39), n	Retained disc (n=36), n	Wrong level disc (n=9), n	Incomplete decompression (n=42), n	Pseudoarthrosis (n=24), n	Total (n=150)
Microdiscectomy	18	12	6	0	6	42
Interlaminar fenestration	12	9	3	3	3	30
Laminectomy	0	0	0	18	6	24
Laminectomy and discectomy	9	15	0	0	3	27
Laminectomy + fusion	0	0	0	21	6	27

Perioperative complications

Of the total 150 patients, perioperative complications were recorded in 69 patients, giving an overall complication rate of 46%. The most common complication was dural tear, which occurred in 24 patients (16%). Wound infection occurred in 18 patients (12%), discitis in nine patients (6%), neurological injury in six patients (4%), and pseudoarthrosis in 12 patients (8%). The postoperative complications following revision lumbar spine surgery are presented in Table 5.

**Table 5:** Perioperative complication

Complication	Frequency (n=69)	Percentage
Dural tear	24/150	16%
Wound infection	18/150	12%
Discitis	9/150	6%
Neurological injury	6/150	4%
Pseudoarthrosis	12/150	8%

Clinical and functional outcomes

Postoperative clinical improvement was observed in a substantial proportion of patients following revision lumbar surgery. At three months, 93 patients (62%) were pain-free. At 12 months, 105 patients (70%) reported pain-free status, and this outcome was maintained at 24 months. Among patients with preoperative neurological deficits, sensory improvement was documented in 15 of 48 patients (31%), and motor improvement occurred in 21 of 39 patients (53%). These sensory and motor improvement rates remained unchanged at 12 and 24 months. Clinical outcomes are summarised in Table 6.

**Table 6:** Postoperative outcome at three, 12, and 24 months

Outcome	3 months	12 months	24 months
Pain free (n=150)	93/150 (62 %)	105/150 (70%)	105/150 (70%)
Sensory improvement (n=48)	15/48 (31%)	15/48 (31%)	15/48 (31%)
Motor improvement (n=39)	21/39 (53%)	21/39 (53%)	21/39 (53%)

Functional outcomes improved significantly during follow-up. The mean VAS score decreased from 7.22 ± 0.89 preoperatively to 3.00 ± 1.27 at three months, 1.47 ± 0.95 at 12 months, and 0.98 ± 0.75 at 24 months. Similarly, the mean ODI score improved from 66.30 ± 9.06 preoperatively to 26.45 ± 9.26 at three months, 11.45 ± 6.55 at 12 months, and 7.77 ± 3.55 at 24 months. The improvements in both VAS and ODI were statistically significant, with p = 0.001 for each measure, supporting sustained postoperative pain relief and functional recovery after revision lumbar surgery. Pain-free status was defined as a VAS pain score of 0 at follow-up. Changes in VAS and ODI scores during follow-up are presented in Table 7.

**Table 7:** Evaluation of ODI and VAS Measurements

Measurement time	ODI	VAS
	Mean ± SD	Mean ± SD
Preoperative	66.30 ± 9.06	7.22 ± 0.89
Postoperative month 3	26.45 ± 9.26	3.0 ± 1.27
Postoperative month 12	11.45 ± 6.55	1.47 ± 0.95
Postoperative month 24	7.77 ± 3.55	0.98 ± 0.75
P-values	0.001	0.001

## Discussion

FBSS remains a challenging condition because its causes are multifactorial and may include recurrent disc herniation, retained disc fragment, incomplete decompression, wrong-level surgery, postoperative instability, pseudoarthrosis, adjacent segment disease, or instrumentation-related failure. Conservative management is generally considered the first-line approach in many patients and may include medication, physical therapy, cognitive behavioural therapy, pain psychology, and other non-operative interventions. Physical therapy may help optimise posture, gait, spinal stability, flexibility, and muscle strength [15,16]. However, nonsurgical treatment is not satisfactory in all patients. In the present study, revision surgery was performed only in patients who had persistent or recurrent symptoms despite conservative treatment and in whom clinical and radiological findings demonstrated a surgically correctable pathology.

The mean age of patients in this study was 41 ± 10.09 years, and most patients were aged between 40 and 60 years. This is comparable to the findings of Assaker and Zairi, who reported that patients with FBSS after disc herniation surgery were generally younger than those who had undergone laminectomy for lumbar stenosis [17]. The relatively younger age in the present study may reflect the predominance of degenerative disc disease and recurrent or residual disc pathology rather than isolated degenerative lumbar stenosis.

There was a male predominance in the present study, with a male-to-female ratio of 1.27:1. Kaner et al. also reported a male predominance in their prospective study of recurrent lumbar disc herniation treated with microdiscectomy and posterior dynamic transpedicular stabilisation, including 23 male and 17 female patients [18]. This male predominance may be related to occupational and mechanical factors, including greater exposure to heavy physical activity, although this association was not specifically analysed in the present study.

In the present study, the diagnosis of FBSS was based on correlation between clinical presentation and radiological findings. MRI and CT were used to identify structural causes of persistent or recurrent symptoms. This approach is important because revision surgery should not be performed unless symptoms, neurological findings, and imaging abnormalities are concordant. Baber and Erdek emphasised that successful management of FBSS depends on identifying a clear aetiology of pain and exhausting conservative measures before considering revision surgery; they also reported that undiagnosed lateral spinal stenosis may account for up to 58% of FBSS cases, highlighting the importance of accurate preoperative diagnosis [19]. In the present study, incomplete decompression was the most common cause of failed previous lumbar surgery, occurring in 42 patients (28%). This was followed by recurrent disc herniation in 39 patients (26%), retained disc fragment in 36 patients (24%), pseudoarthrosis or instrumentation-related failure in 24 patients (16%), and wrong-level surgery in nine patients (6%).

Other studies have also identified foraminal or canal stenosis, recurrent disc herniation, instability, degenerative disc disease, and non-union of spinal fusion as important structural causes of FBSS [19,20]. Durand et al. described foraminal stenosis, degenerative disc disease, recurrent disc herniation, and non-union of spinal fusion as the main aetiologies investigated in patients with FBSS, although exact percentages for each cause were not provided in the accessible abstract [20]. MRI remains the main imaging modality in most cases, while CT or CT myelography may be useful when bony pathology, instrumentation-related complications, pseudoarthrosis, or residual stenosis are suspected [19,20].

The relationship between the type of previous surgery and the cause of failure was clinically important. Recurrent disc herniation was most observed after previous microdiscectomy and interlaminar decompression/fenestration. Among patients who had previous microdiscectomy, 24 of 42 patients had recurrent disc herniation, while among patients who had previous interlaminar decompression/fenestration, 12 of 30 patients had recurrent disc herniation. This supports the view that recurrent disc herniation remains an important cause of failed outcome after disc surgery. In comparison, Ali et al. reported recurrent disc herniation at the same level in nine patients (29%), wrong-level surgery in five patients (21.1%), instability in six patients (19.4%), root injury in three patients (9.7%), fibrosis in three patients (9.7%), adhesive arachnoiditis in two patients (6.5%), canal stenosis in one patient (3.2%), and unknown cause in two patients (6.5%) [21]. El Shazly et al. also studied recurrent lumbar disc herniation and compared three surgical options, including discectomy alone, discectomy with transforaminal lumbar interbody fusion, and discectomy with posterolateral fusion [22].

In contrast, incomplete decompression was more frequently associated with previous laminectomy and laminectomy with fusion. In the present study, 18 of 24 patients with previous laminectomy and 21 of 27 patients with previous laminectomy and fusion had incomplete decompression. This finding highlights the importance of adequate decompression at the index operation, careful assessment of lateral recess and foraminal stenosis, and appropriate preoperative imaging before surgery.

Revision lumbar surgery is technically more challenging than primary surgery because of distorted anatomy, unclear tissue planes, epidural fibrosis, and perineural scarring. These factors may increase operative time, blood loss, and complication rates. Bono and Brick reported that revision surgery for lumbar stenosis is associated with longer operative time, greater technical difficulty, and a higher potential complication rate than primary surgery; however, clinical success can still be achieved in well-selected patients [23].

In the present study, postoperative complications occurred in 69 patients (46%). The most common complication was dural tear, occurring in 24 patients (16%). This is consistent with the known increased risk of dural tear during revision surgery, particularly during scar dissection. Tafazal and Sell, in a prospectively collected series of 1549 lumbar spine operations, reported dural tear rates of 3.5% for primary discectomy, 8.5% for spinal stenosis surgery, and 13.2% for revision discectomy [24]. This supports the observation that revision procedures carry a higher risk of incidental durotomy than primary lumbar surgery.

Wound infection occurred in 18 patients (12%), while discitis occurred in nine patients (6%). Discitis was reported following instrumented fusion. Most cases were treated conservatively; however, three patients required screw removal because of infection and subsequently developed pseudoarthrosis with persistent back pain. This emphasises the importance of infection prevention, careful wound care, and close postoperative monitoring, particularly in patients undergoing instrumented fusion.

Postoperative neurological deficit occurred in six patients (4%). These cases presented mainly with paraesthesia and numbness and were managed conservatively with analgesia and supportive treatment. Neurological injury after lumbar decompression is generally uncommon, but the risk may increase during revision surgery because of adhesions, nerve root tethering, and difficult dissection around scar tissue.

Clinical outcomes improved after revision surgery in a substantial proportion of patients. At three months, 93 patients (62%) were pain-free or had major pain relief. At 12 and 24 months, 105 patients (70%) reported pain-free status or major pain relief. This is consistent with previous reports showing that revision surgery can achieve satisfactory outcomes in selected patients. El Shazly et al. reported a mean overall recovery rate of 87.2% and a satisfactory outcome rate of 88.9% in patients treated surgically for recurrent lumbar disc herniation [22]. Other reports have suggested that excellent or good outcomes after repeated lumbar discectomy range from approximately 70% to 89%, although outcomes are generally slightly lower than those after primary surgery [25].

Sensory improvement was documented in 15 of 48 patients (31%), and motor improvement in 21 of 39 patients (53%). Functional outcomes also improved significantly, with marked reductions in both VAS and ODI scores at three, 12, and 24 months. The mean VAS score improved from 7.22 ± 0.89 preoperatively to 0.98 ± 0.75 at 24 months. Similarly, the mean ODI score improved from 66.30 ± 9.06 preoperatively to 7.77 ± 3.55 at 24 months. These findings suggest that revision surgery can provide sustained pain relief and functional improvement when patients are carefully selected and when surgery is directed toward a clear structural cause.

Strengths and limitations

The strengths of this study include a relatively large single-centre cohort, detailed assessment of the causes and procedures related to revision lumbar surgery, and 24-month clinical and functional follow-up using validated VAS and ODI measures. However, the retrospective single-centre design may limit generalisability and introduce selection and documentation bias. The absence of a conservatively managed control group limits comparative conclusions regarding the effectiveness of revision surgery. In addition, the cohort included heterogeneous pathologies and procedures, subgroup outcomes were not analysed separately for each revision technique, and outcome assessment relied on routinely recorded clinical data. Psychosocial factors, work status, and broader quality-of-life measures were not assessed. Therefore, the findings should be interpreted cautiously and confirmed in prospective multicentre controlled studies using standardised outcome assessment.

## Conclusions

In this selected single-centre cohort of patients with radiologically confirmed, surgically correctable pathology, revision lumbar surgery was associated with sustained improvements in pain and disability over 24 months. The most common causes of failed previous lumbar surgery were incomplete decompression, recurrent disc herniation, retained disc fragment, and pseudoarthrosis or instrumentation-related failure. Accurate clinical-radiological correlation and careful patient selection are essential to identify the cause of symptoms and guide the appropriate revision procedure. However, revision surgery remains technically demanding because of scar tissue, altered anatomy, and the increased risk of complications, particularly dural tear. Therefore, although meaningful pain relief and functional improvement were observed, the substantial complication rate and partial neurological recovery indicate that outcomes were mixed and should not be generalised to the broader FBSS population.
